# The store-operated Ca^2+^ channel Orai1α is required for agonist-evoked NF-κB activation by a mechanism dependent on PKCβ2

**DOI:** 10.1016/j.jbc.2023.102882

**Published:** 2023-01-07

**Authors:** Joel Nieto-Felipe, Jose Sanchez-Collado, Isaac Jardin, Gines M. Salido, Jose J. Lopez, Juan A. Rosado

**Affiliations:** Department of Physiology (Cellular Physiology Research Group), Institute of Molecular Pathology Biomarkers (IMPB), University of Extremadura, Caceres, Spain

**Keywords:** Orai1α, Orai1β, store-operated Ca^2+^ entry, NF-κB, PKCβ2, AKAR, AKAR, AKAP79 association region, AM, acetoxymethyl ester, BSA, bovine serum albumin, CCh, carbachol, CMV, cytomegalovirus, eGFP, enhanced GFP, HEK-293, human embryonic kidney 293 cell line, IgG, immunoglobulin G, IKK, IκB kinase, qRT–PCR, quantitative RT–PCR, RBX, ruboxistaurin, SERCA, sarcoplasmic/endoplasmic reticulum Ca^2+^-ATPase, SOCE, store-operated Ca2+ entry, STIM, stromal interaction molecule, TBST, Tris-buffered saline with 0.1% Tween-20, TG, thapsigargin, TK, thymidine kinase, TKO, triple KO

## Abstract

Store-operated Ca^2+^ entry is a ubiquitous mechanism for Ca^2+^ influx in mammalian cells that regulates a variety of physiological processes. The identification of two forms of Orai1, the predominant store-operated channel, Orai1α and Orai1β, raises the question whether they differentially regulate cell function. Orai1α is the full-length Orai1, containing 301 amino acids, whereas Orai1β lacks the N-terminal 63 amino acids. Here, using a combination of biochemistry and imaging combined with the use of human embryonic kidney 293 KO cells, missing the native Orai1, transfected with plasmids encoding for either Orai1α or Orai1β, we show that Orai1α plays a relevant role in agonist-induced NF-κB transcriptional activity. In contrast, functional Orai1β is not required for the activation of these transcription factors. The role of Orai1α in the activation of NF-κB is entirely dependent on Ca^2+^ influx and involves PKCβ activation. Our results indicate that Orai1α interacts with PKCβ2 by a mechanism involving the Orai1α exclusive AKAP79 association region, which strongly suggests a role for AKAP79 in this process. These findings provide evidence of the role of Orai1α in agonist-induced NF-κB transcriptional activity and reveal functional differences between Orai1 variants.

Agonist-evoked intracellular Ca^2+^ mobilization is a finely regulated process aimed at the generation of spatiotemporal Ca^2+^ signals that match the strength of agonist stimulation. Among the mechanisms involved in intracellular Ca^2+^ homeostasis, store-operated Ca^2+^ entry (SOCE) is a major mechanism for agonist-evoked Ca^2+^ mobilization. SOCE plays a relevant functional role supporting a variety of cellular processes from neurotransmitter release to gene transcription (1, 2, 3). Phospholipase C-coupled receptor occupation results in the generation of inositol trisphosphate, which, upon binding to the inositol trisphosphate receptors in the membrane of the endoplasmic reticulum, leads to Ca^2+^ efflux from the intracellular Ca^2+^ stores. Ca^2+^ store depletion triggers a conformational change of the stromal interaction molecule (STIM) proteins, the endoplasmic reticulum Ca^2+^ sensor, allowing store-dependent activation of Orai channels in the plasma membrane (4, 5, 6, 7, 8). Orai1 and its paralogs in mammalian cells, Orai2 and Orai3, have been reported to heteromultimerize to fit the strength of agonist stimulation to precise spaciotemporal Ca^2+^ signals (9, 10).

The NF-κB is a transcription factor found in nearly all mammalian cells. NF-κB is one of the key regulators of inflammatory immune responses and, in addition, it is involved in a number of cellular functions, including survival, synaptic plasticity, cell proliferation, and differentiation (11, 12, 13). Inactive NF-κB is located in the cytosol complexed with the inhibitor IκB. The primary mechanism for regulating NF-κB involves serine phosphorylation and proteasomal degradation of IκB that releases active, dimeric, NF-κB, which translocates to the nucleus and binds to NF-κB-responsive genes (14). This signaling pathway occurs primarily by activation of the IκB kinase (IKK) (15). Calcium influx *via* the T-type Ca^2+^ channel Ca_v_3.3 has been reported to lead to activation of PKCβ, a Ca^2+^- and diacylglycerol-regulated conventional PKC isoform, which in turn activates NF-κB through the classical IKK–IκB pathway resulting in increased proliferation (16) and, more recently, T cell receptor–evoked IKK activation has been reported to require Ca^2+^ influx *via* STIM1-dependent Orai1 channels (17).

In mammalian cells, two Orai1 variants have been identified, the long form, Orai1α is the full-length Orai1 containing 301 amino acids, whereas the short variant, Orai1β, arises from the same transcript by alternative translation initiation from a methionine at position 64, or even 71, in Orai1α (18). Several biophysical and functional differences between both variants have been reported. While both variants support with similar efficiency, the store-operated current *I*_crac_, the arachidonate-regulated current, *I*_arc_, is only supported by Orai1α (19), and the participation of Orai1β in *I*_soc_, the current involving STIM1, Orai1, and TRPC1, seems to be cell specific (20). Furthermore, Orai1α is more sensitive to Ca^2+^-dependent inactivation (19), which might involve specific phosphorylation of Orai1α at Ser34 in an adenylyl cyclase 8–dependent manner (2). Here, we show that Orai1α plays an essential role in agonist-induced NF-κB activation, whereas Orai1β does not seem to be required for the activation of these transcription factors. The activation of the transcriptional activity of NF-κB by Orai1α is a mechanism that entirely depends on Ca^2+^ influx and involves the Ca^2+^-regulated PKCβ isoform. The Orai1α exclusive AKAR (AKAP79 association region containing amino acids 39–59 within the N terminus of Orai1α) plays an essential role in the interaction with PKCβ, thus suggesting a role for AKAP79 in this process. These findings provide evidence of functional differences between Orai1 variants in the activation of NF-κB transcriptional activity.

## Results

### Orai1α, but not Orai1β, supports carbachol-evoked NF-κB activation

**Figure 1:**
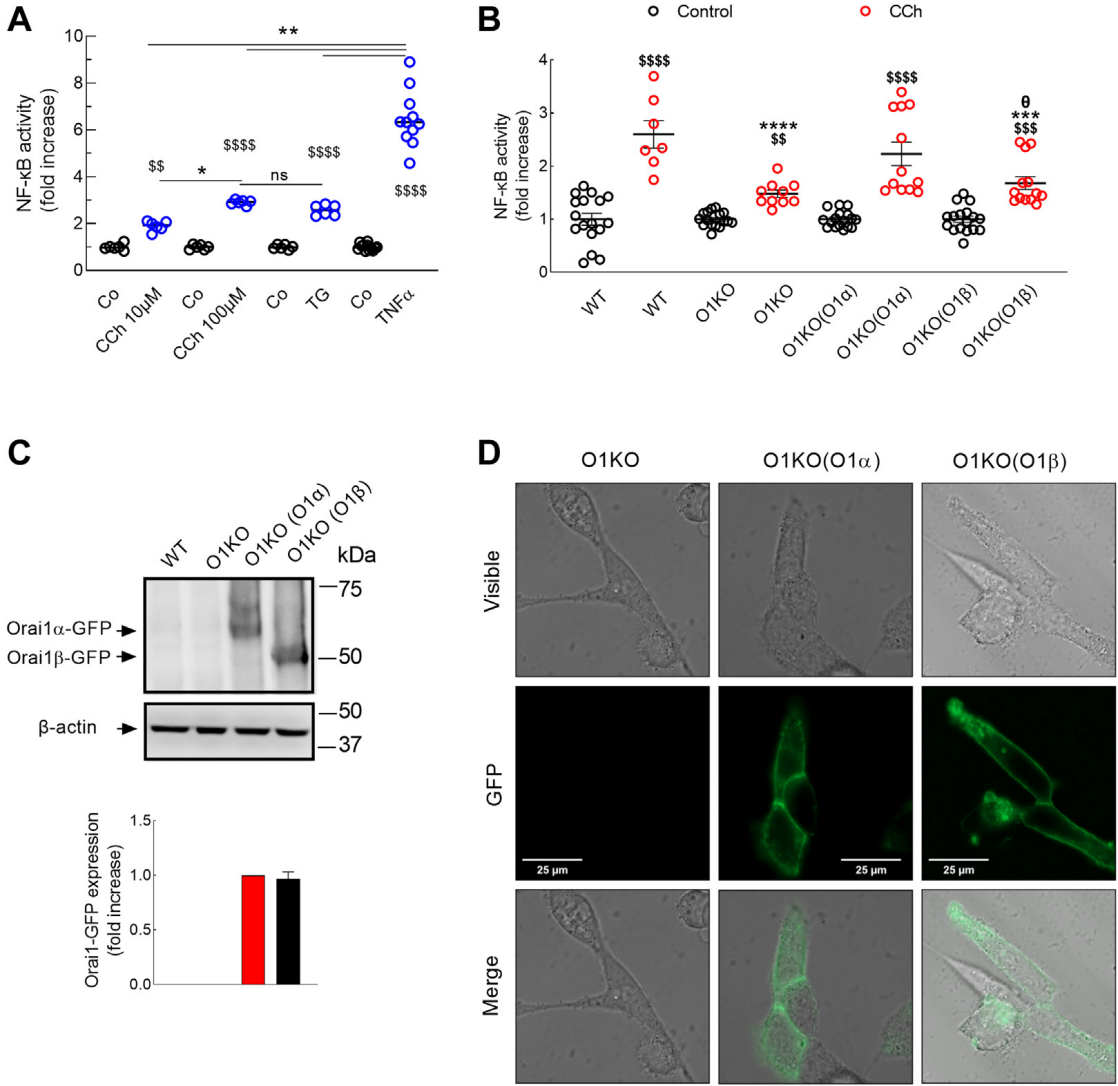
**Orai1α rescues CCh-evoked NF-κB activation in Orai1-KO cells.***A,* WT HEK-293 cells were transfected with pNL3.2.NFkB-RE[NlucP/NF-kB-RE/Hygro]. Forty-eight hours later, cells were suspended in HBS containing 1 mM Ca^2+^ and then stimulated for 5 h with 10 or 100 μM CCh, 1 μM TG, 20 ng/ml TNFα, or the vehicle (control) and lysed. Luciferase activity of the lysates was measured using a Nano-Glo Luciferase Reporter Assay System, according to the manufacturer’s instructions. From *left* to *right*, *n* = 6, 6, 6, 6, 6, 6, 12, and 11; n values correspond to separate experiments. The luminescence in relative light units (RLUs) of unstimulated WT was 615,487 ± 32,239. Scatter plots are represented as mean ± SEM and were statistically analyzed using Kruskal–Wallis test with multiple comparisons (Dunn's test). ∗*p* < 0.05, ∗∗*p* < 0.01. ^$$^*p* < 0.01, and ^$$$$^*p* < 0.0001 as compared with their respective control (untreated) cells. *B,* WT HEK-293 cells (WT), Orai1-KO HEK-293 cells (O1KO), and Orai1-KO HEK-293 cells expressing either Orai1α (O1KO(O1α)) or Orai1β (O1KO(O1β)) were transfected with pNL3.2.NFkB-RE[NlucP/NF-kB-RE/Hygro]. Forty-eight hours later, cells were suspended in HBS containing 1 mM Ca^2+^ and then stimulated for 5 h with 100 μM CCh or the vehicle (control) and lysed. Luciferase activity of the lysates was measured using a Nano-Glo Luciferase Reporter Assay System, according to the manufacturer’s instructions. From *left* to *right*, n = 16, 7, 16, 10, 16, 12, 16, and 13; n values correspond to separate experiments. The luminescence in RLUs of unstimulated WT, O1KO, O1KO(O1α), and O1KO(O1β) HEK-293 cells were 632,425 ± 59,231, 616,660 ± 73,069, 609,718 ± 19,914, and 618,231 ± 34,847, respectively). *C,* WT, O1KO, O1KO(O1α), and O1KO(O1β) HEK-293 cells were lysed and then subjected to 10% SDS-PAGE and Western blotting with the anti-Orai1 antibody, as described in the Experimental procedures section. Membranes were reprobed with the anti-β-actin antibody for protein loading control. Molecular masses indicated on the *right* were determined using molecular-mass markers run in the same gel. Blots are representative of four separate experiments, and intensity of Orai1-GFP bands was normalized to β-actin and quantified (*bar graph*). Scatter plots are represented as mean ± SEM and statistically analyzed using Kruskal–Wallis test with multiple comparisons (Dunn's test). ∗∗∗*p* < 0.001 and ∗∗∗∗*p* < 0.0001 as compared with CCh-treated WT HEK-293 cells. ^$$^*p* < 0.01, ^$$$^*p* < 0.001, and ^$$$$^*p* < 0.0001 as compared with their respective control (untreated) cells. ^θ^*p* < 0.05 as compared with CCh-treated O1KO(O1α) HEK-293 cells. *D,* representative confocal images of Orai1α-GFP or Orai1β-GFP expressed in Orai1-KO HEK-293 cells. The scale bar represents 25 μm. CCh, carbachol; HBS, Hepes-buffered saline; HEK-293, human embryonic kidney 293 cell line; TG, thymidine kinase; TNFα, tumor necrosis factor alpha.

NF-κB has been reported to play relevant functional roles, including cell proliferation, differentiation, and survival (13). Human embryonic kidney 293 (HEK-293) cells were treated for 5 h with different agonists, lysed, and luciferase activity was measured using a Nano-Glo Luciferase Reporter Assay System. As shown in Figure 1*A*, treatment with 10 or 100 μM carbachol (CCh) significantly (*p* < 0.01 and *p* < 0.0001, respectively) enhanced NF-κB activity over the pretreatment level in a concentration-dependent manner (*p* < 0.05). Similar results were observed when cells were stimulated with the sarcoplasmic/endoplasmic reticulum Ca2+-ATPase (SERCA) inhibitor thapsigargin (TG) (Fig. 1*A*; *p* < 0.0001). These data indicate that agonist stimulation and Ca^2+^ store depletion evoke NF-κB activity in HEK-293 cells with similar efficacy. As a positive control, we stimulated cells with tumor necrosis factor alpha (20 ng/ml), which exhibits a significantly greater effect than CCh and TG (Fig. 1*A*; *p* < 0.01). Transcriptional activity of NF-κB after cell treatment with TG or tumor necrosis factor alpha was confirmed by the mRNA quantification of COX-2, an NF-κB target gene, using a quantitative RT–PCR (qRT–PCR) analysis (Fig. S1).

We have further explored the functional role of the Orai1 variants, Orai1α and Orai1β, on agonist-induced NF-κB activation using WT HEK-293 cells, Orai1-KO HEK-293 cells (O1KO), and Orai1-KO HEK-293 cells expressing either Orai1α (O1KO(O1α)) or Orai1β (O1KO(O1β)). Cells were stimulated for 5 h with CCh (100 μM) or the vehicle (control) (Fig. 1*B*), and NF-κB activity was determined as described in the Experimental procedures section. Expression of Orai1 variants was demonstrated by Western blotting using a specific anti-Orai1 (C-terminal) antibody (Fig. 1*C*). As depicted in Figure 1*B*, treatment of WT-HEK-293 cells with CCh significantly increased NF-κB activity as compared with untreated cells. Interestingly, CCh-evoked increase in NF-κB activity was significantly reduced in O1KO cells (Fig. 1*B*; *p* < 0.0001), which strongly supports a role for Orai1 in NF-κB activation, although a significant response to CCh still remains in O1KO cells (Fig. 1*B*; *p* < 0.05). To further identify the Orai1 variant involved in this process, O1KO HEK cells were transfected with either Orai1α or Orai1β using expression plasmids carrying a thymidine kinase (TK) promoter, which leads to a protein expression level significantly smaller than plasmids with the cytomegalovirus (CMV) promoter (19). Transfected cells were analyzed for Orai1 expression by confocal microscopy. Cells transfected with either Orai1α or Orai1β confirmed fluorescence labeling confined exclusively at/by the plasma membrane (Fig. 1*D*). As shown in Figure 1*B*, expression of Orai1α rescues CCh-evoked NF-κB activation; by contrast, expression of Orai1β did not significantly enhance CCh-induced response as compared with O1KO cells and was significantly less effective than Orai1α (*p* < 0.05; Fig. 1*B*). These findings indicate that Orai1α, but not Orai1β, supports CCh-evoked NF-κB activation.

**Figure 2:**
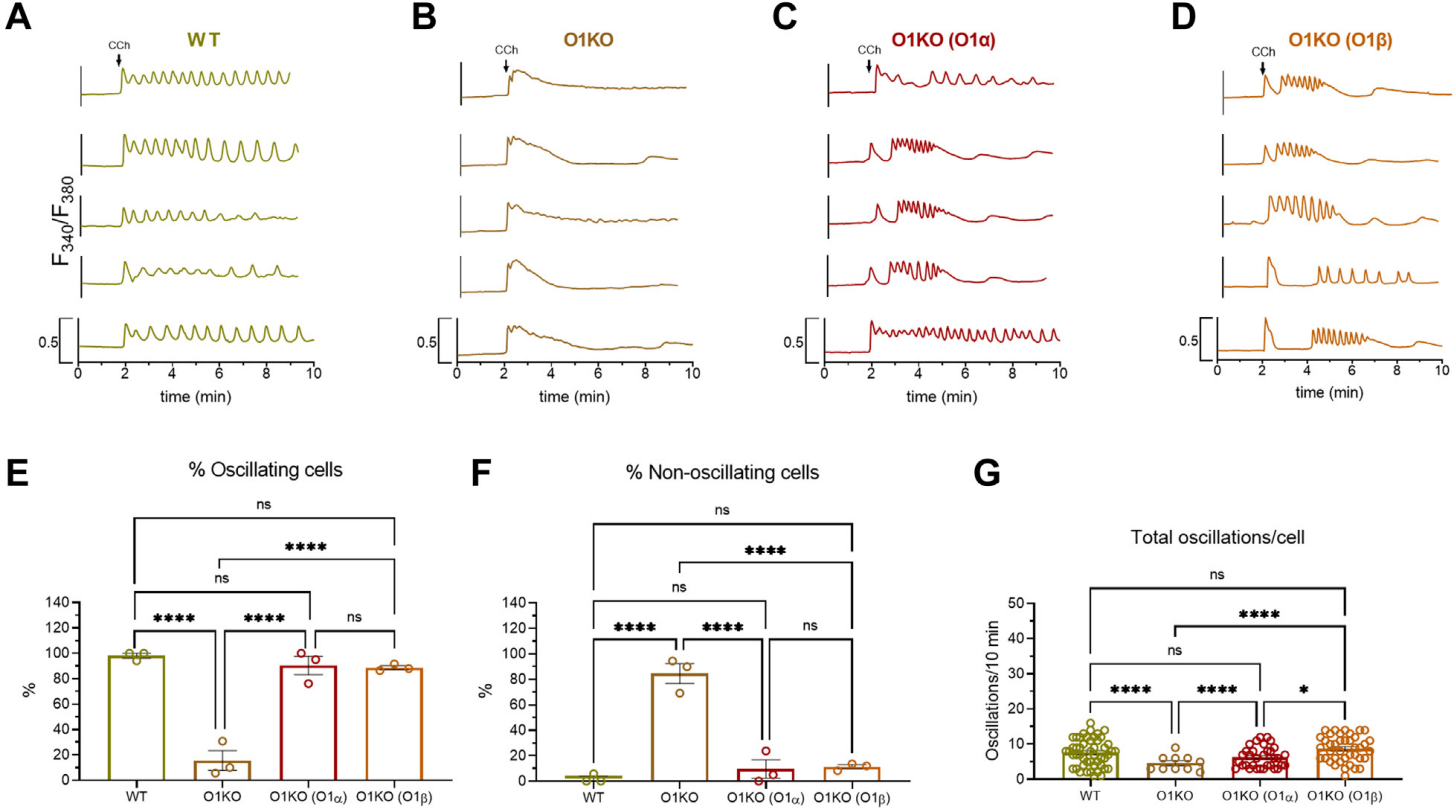
**Role of Orai1α and Orai1β in Ca**^**2+**^**mobilization induced by high concentration of carbachol (CCh).***A*–*D,* representative Ca^2+^ mobilization in response to 100 μM CCh measured using fura-2 in WT HEK-293 cells (WT), Orai1-KO HEK-293 cells (O1KO), and Orai1-KO HEK-293 cells expressing either Orai1α (O1K(O1α)) or Orai1β (O1KO(O1β)), as described. Cells were superfused with HBS containing 1 mM Ca^2+^ and stimulated with 100 μM CCh at 2 min (indicated by *arrow*). Representative traces from five cells/condition were chosen to represent the datasets. *E–G,* quantification of the percentage of oscillating cells (*E*), percentage of nonoscillating cells (*F*), and total oscillations/cell in 8 min (*G*) for data presented in *A–D* (for *E* and *F*, n = 3; n values correspond to independent experiments; for *G*, from *left* to *right*, n = 50, 10, 45, and 42; n values correspond to individual cells). Scatter plots are represented as mean ± SEM and were statistically analyzed using Kruskal–Wallis test with multiple comparisons (Dunn's test). ∗*p* < 0.05 and ∗∗∗∗*p* < 0.0001). HBS, Hepes-buffered saline; HEK-293, human embryonic kidney 293 cell line.

**Figure 3:**
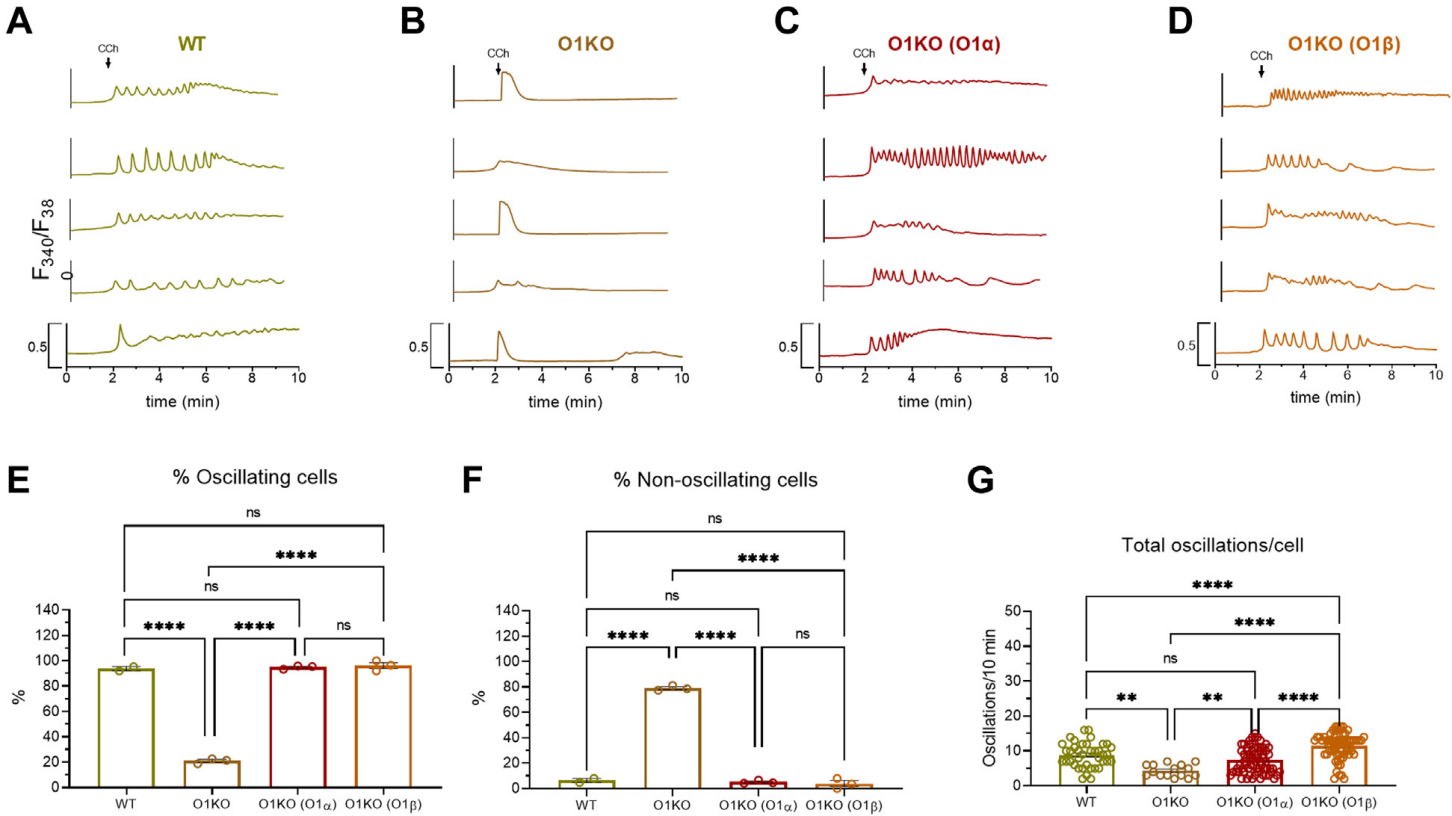
**Role of Orai1α and Orai1β in Ca**^**2+**^**mobilization induced by low concentration of carbachol (CCh).***A–D,* representative Ca^2+^ mobilization in response to 10 μM CCh measured in fura 2-loaded WT HEK-293 cells (WT), Orai1-KO HEK-293 cells (O1KO), and Orai1-KO HEK-293 cells expressing either Orai1α (O1KO(O1α)) or Orai1β (O1KO(O1β)), as described. Cells were superfused with HBS containing 1 mM Ca^2+^ and stimulated with 10 μM CCh at 2 min (indicated by *arrow*). Representative traces from five cells/condition were chosen to represent the datasets. *E–G,* quantification of the percentage of oscillating cells (*E*) and nonoscillating cells (*F*) and total oscillations/cell in 8 min (*G*) for data presented in *A*–*D* (for *E* and *F*, n = 3; n values correspond to independent experiments; for *H*, from *left* to *right*, n = 39, 15, 70, and 67; n values correspond to individual cells). Scatter plots are represented as mean ± SEM and were statistically analyzed using Kruskal–Wallis test with multiple comparisons (Dunn's test). ∗∗*p* < 0.01 and ∗∗∗∗*p* < 0.0001. HBS, Hepes-buffered saline; HEK-293, human embryonic kidney 293 cell line.

Next, we explored the role of Orai1α and Orai1β on CCh-induced Ca^2+^ mobilization in WT HEK-293 cells, O1KO HEK-293 cells, and O1KO HEK-293 cells expressing either Orai1α or Orai1β. Traces from five representative cells are depicted in Figure 2, *A*–*D*. In the presence of 1 mM extracellular Ca^2+^, about 98% of WT HEK-293 cells responded to CCh with regenerative Ca^2+^ oscillations (Fig. 2, *A* and *E*), with an average of 7.4 ± 0.5 oscillations/8 min (Fig. 2*G*). The cells that did not oscillate responded with a sustained plateau (Fig. 2*F*). In O1KO cells, where Ca^2+^ entry is impaired, only 15% of the cells responded with Ca^2+^ oscillations after stimulation with CCh, with an average of 4.6 ± 0.6 oscillations/8 min (Fig. 2*G*). The remaining cells responded with transient Ca^2+^ signals because of Ca^2+^ release from the intracellular stores. As a result, the magnitude of Ca^2+^ mobilization induced by CCh was significantly greater in WT, which reveals the role of Orai1 in CCh-induced Ca^2+^ signals (Fig. S2; *p* < 0.0001). When O1KO cells were transfected with Orai1α or Orai1β expression plasmid, the percentage of cells that responded with regenerative Ca^2+^ oscillations enhanced to 90% and 88%, for Orai1α and Orai1β, respectively (Fig. 2*E*; *p* < 0.0001). Furthermore, expression of Orai1α or Orai1β significantly enhanced the average number of oscillations per cell as compared with O1KO cells (6.3 ± 0.5 and 8.4 ± 0.6 oscillations/8 min for Orai1α and Orai1β, respectively; Figure 2*G*; *p* < 0.0001), and expression of Orai1β displays a significantly higher oscillation frequency than Orai1α (Fig. 2*G*; *p* < 0.05), as previously reported (2). The magnitude of Ca^2+^ mobilization upon treatment with CCh was significantly greater in Orai1α- and Orai1β-expressing cells than in O1KO cells (Fig. S2; *p* < 0.0001 and *p* < 0.01 for Orai1α and Orai1β, respectively). Similar results were observed when cells were stimulated with a low agonist concentration. As shown in Figure 3, most cells responded with Ca^2+^ oscillations upon stimulation with 10 μM CCh. O1KO cells predominantly responded with a transient increase in [Ca^2+^]_i_ as a result of Ca^2+^ release from the intracellular store, and interestingly, expression of Orai1α or Orai1β restored the oscillatory pattern in HEK-293 cells. It is important to note that cells expressing Orai1β exhibited a significantly greater number of oscillations than those expressing Orai1α, which is consistent with its reported smaller sensitivity to Ca^2+^-dependent inactivation (19). Therefore, expression of either Orai1α or Orai1β rescued the pattern of CCh-evoked Ca^2+^ mobilization observed in WT cells.

**Figure 4:**
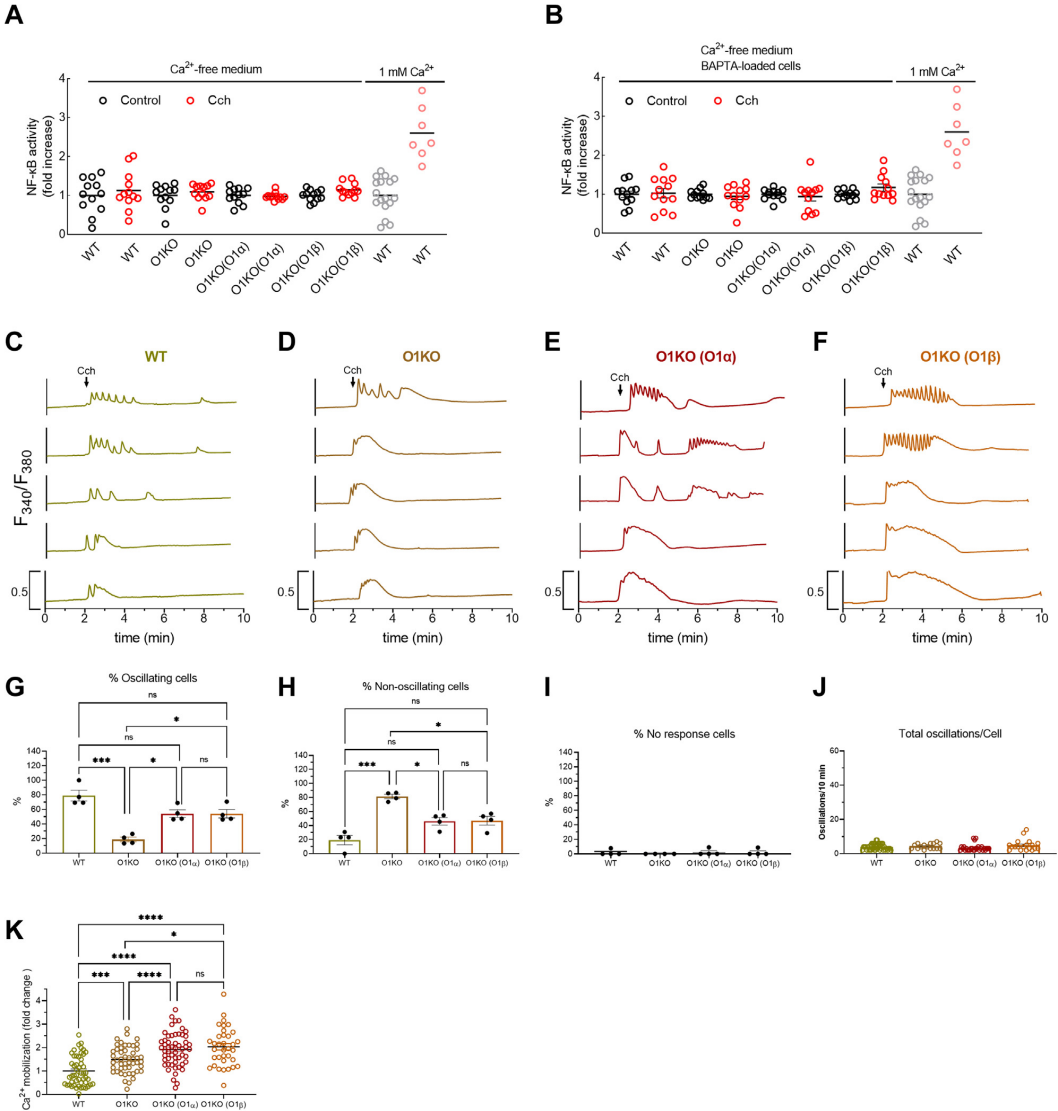
**Role of Ca**^**2+**^**entry in carbachol (CCh)-evoked NF-κB activation.***A* and *B,* WT HEK-293 cells (WT), Orai1-KO HEK-293 cells (O1KO), and Orai1-KO HEK-293 cells expressing either Orai1α (O1KO(O1α)) or Orai1β (O1KO(O1β)) were transfected with pNL3.2.NFkB-RE[NlucP/NF-kB-RE/Hygro]. Forty-eight hours later, cells were suspended in a Ca^2+^-free HBS (100 μM EGTA added) (*A*), were loaded with dimethyl-BAPTA and suspended in a Ca^2+^-free HBS (*B*), or were suspended in HBS containing 1 mM Ca^2+^ (*A* and *B gray and light red dots*), as indicated. Cells were then stimulated for 5 h with 100 μM CCh or the vehicle (control) and lysed. Luciferase activity of the lysates was measured as described in the Experimental procedures section. For *A* and *B*, n = 12; n values correspond to separate experiments. The luminescence in relative light units (RLUs) of unstimulated WT, O1KO, O1KO(O1α), and O1KO(O1β) HEK-293 cells suspended in a Ca^2+^-free medium were 607,412 ± 36,588, 602,587 ± 39,898, 612,289 ± 33,251, and 599,252 ± 30,244, respectively, the RLUs of BAPTA-loaded unstimulated WT, O1KO, O1KO(O1α), and O1KO(O1β) HEK-293 cells suspended in a Ca^2+^-free medium were 601,522 ± 41,252, 598,230 ± 37,752, 605,211 ± 38,904, and 604,287 ± 37,748, respectively, and the RLUs of resting WT cells suspended in a medium containing 1 mM Ca^2+^ was 632,425 ± 59,231). *C–F,* representative Ca^2+^ mobilization in response to 100 μM CCh measured using fura-2 in WT HEK-293 cells (WT), Orai1-KO HEK-293 cells (O1KO), and Orai1-KO HEK-293 cells expressing either Orai1α (O1KO(O1α)) or Orai1β (O1KO(O1β)), as described. Cells were superfused with Ca^2+^-free HBS and stimulated with 100 μM CCh at 2 min (indicated by *arrow*). Representative traces from five cells/condition were chosen to represent the datasets. *G–J,* quantification of the percentage of oscillating cells (*G*), percentage of nonoscillating cells (*H*), percentage of nonresponding cells (*I*), and total oscillations/cell in 8 min (*J*) for data presented in *C–F* (for *G* to *I*, n = 4; n values correspond to independent experiments; for *J*, from *left* to *right*, n = 41, 22, 29, and 19; n values correspond to individual cells). *K,* quantification of Ca^2+^ mobilization for all the conditions from *C* to *F* estimated in all the cells. Scatter plots are represented as mean ± SEM and were statistically analyzed using Kruskal–Wallis test with multiple comparisons (Dunn's test) to WT HEK-293 cells (∗∗*p* < 0.01 and ∗∗∗*p* < 0.001) or O1KO HEK-293 cells (∗*p* < 0.05, ∗∗*p* < 0.01, ∗∗∗*p* < 0.001, and ∗∗∗∗*p* < 0.0001). HBS, Hepes-buffered saline; HEK-293, human embryonic kidney 293 cell line.

To ascertain the role of Ca^2+^ influx and Ca^2+^ mobilization in Orai1α-dependent NF-κB activation, we performed a series of experiments in the absence of extracellular Ca^2+^ and in cells loaded with the Ca^2+^ chelator dimethyl BAPTA to prevent [Ca^2+^]_i_ rises (Fig. 4, *A* and *B*). CCh-induced Ca^2+^ mobilization in WT HEK-293 cells, O1KO HEK-293 cells, and O1KO HEK-293 cells expressing Orai1α or Orai1β in a Ca^2+^-free medium is shown in Figure 4, *C*–*K*. Traces from five representative cells are shown in Figure 4, *C*–*F*. An average of 78, 18, 54, and 54% of WT, O1KO, and O1KO cells expressing either Orai1α or Orai1β stimulated with CCh in a Ca^2+^-free medium responded with Ca^2+^ oscillations because of periodic discharge of stored Ca^2+^ (Fig. 4, C–*F* and *G*), with an average of 3.9 ± 0.2, 4.3 ± 0.3, 3.3 ± 0.3, and 4.9 ± 0.7 oscillations/8 min (Fig. 4*J*). All the cells that did not oscillate responded with single Ca^2+^ transients (Fig. 4*H*). The magnitude of Ca^2+^ mobilization was slightly greater in cells expressing Orai1 variants (Fig. 4*K*; *p* < 0.05). As shown in Figure 4*A*, in the absence of extracellular Ca^2+^, CCh was unable to enhance NF-κB activity under all conditions tested, and similar results were observed in BAPTA-loaded cells stimulated in a Ca^2+^-free medium (Fig. 4*B*). In BAPTA-loaded cells, suspended in a Ca^2+^-free medium, treatment with CCh was unable to induce Ca^2+^ mobilization (Fig. S3) and, as expected, CCh was without effect on NF-κB activation at all the conditions assessed (Fig. 4*B*). These findings strongly support an essential role for Ca^2+^ influx in CCh-evoked NF-κB activation in these cells.

### TRPC1 and Orai3 mediate CCh-evoked NF-κB activation in Orai1-KO cells

**Figure 5:**
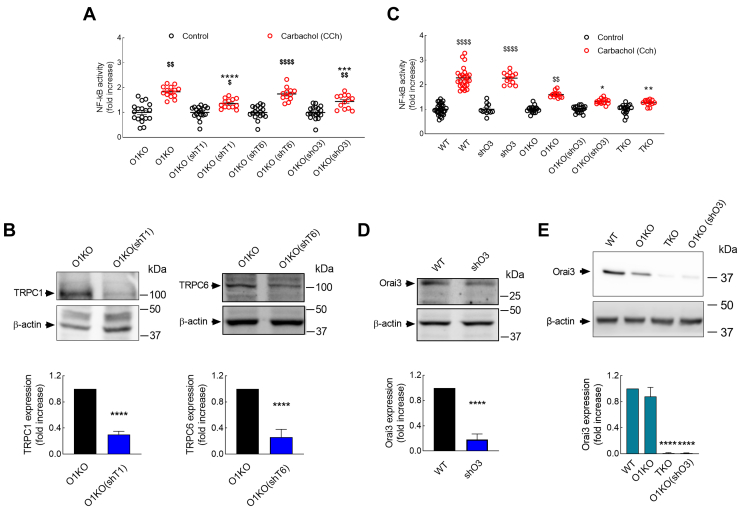
**TRPC1 and Orai3 are involved in carbachol (CCh)-evoked NF-κB activation in Orai1-KO cells.***A,* Orai1-KO HEK-293 cells (O1KO) transfected with either shTRPC1 (O1KO (shT1)), shTRPC6 (O1KO (shT6)), shOrai3 (O1KO (shO3)), or scramble plasmid (O1KO) were transfected with pNL3.2.NFkB-RE[NlucP/NF-kB-RE/Hygro]. Forty-eight hours later, cells were suspended in HBS containing 1 mM Ca^2+^ and then stimulated for 5 h with 100 μM CCh or the vehicle (control) and lysed. Luciferase activity of the lysates was measured as described in the Experimental procedures section. The luminescence in relative light units (RLUs) of unstimulated O1KO, O1KO(shT1), O1KO(shT6), and O1KO(shO3) HEK-293 cells were 598,259 ± 47,521, 601,025 ± 51,248, 599,987 ± 32,581, and 601,277 ± 39,985, respectively). *B,* O1KO, O1KO (shT1), and O1KO (shT6) HEK-293 cells were lysed and then subjected to 10% SDS-PAGE and Western blotting with anti-TRPC1 (*left panels*) or anti-TRPC6 (*right panels*) antibody. Membranes were reprobed with the anti-β-actin antibody for protein loading control. Molecular masses indicated on the *right* were determined using molecular-mass markers run in the same gel. Blots are representative of four separate experiments, and intensity of TRPC1 and TRPC6 bands was normalized to β-actin and quantified (*bar graph*). *C,* WT HEK-293 cells (WT), WT cells transfected with shOrai3 (shO3), O1KO cells, O1KO cells transfected with shOrai3 (O1KO (shO3)), and Orai1/Orai2/Orai3 triple-KO cells (TKO) transfected with pNL3.2.NFkB-RE[NlucP/NF-kB-RE/Hygro]. Forty-eight hours later, cells were suspended in HBS containing 1 mM Ca^2+^ and stimulated for 5 h with 100 μM CCh or the vehicle (control) and lysed. Luciferase activity was measured as described in the Experimental procedures section. The luminescence in RLUs of unstimulated WT, O1KO, O1KO(shO3), and TKO HEK-293 cells were 610,982 ± 48,875, 607,588 ± 47,510, 592,115 ± 37,514, and 597,323 ± 37,899, respectively. *D,* WT HEK-293 cells (WT) and WT cells transfected with shOrai3 (shO3) were lysed and subjected to 10% SDS-PAGE and Western blotting with anti-Orai3 antibody. Membranes were reprobed with the anti-β-actin antibody for protein loading control. Molecular masses indicated on the *right* were determined using molecular-mass markers run in the same gel. Blots are representative of four separate experiments, and intensity of Orai3 bands was normalized to β-actin and quantified (*bar graph*). *E,* WT, O1KO, TKO, and O1KO (shO3) HEK-293 cells were lysed and then subjected to 10% SDS-PAGE and Western blotting with anti-Orai3. Membranes were reprobed with the anti-β-actin antibody for protein loading control. Molecular masses indicated on the *right* were determined using molecular-mass markers run in the same gel. Blots are representative of four separate experiments, and intensity of Orai3 bands was normalized to β-actin and quantified (*bar graph*). For *A,* from *left* to *right*, n = 18, 14, 18, 13, 18, 12, 18, and 13; for *C,* from *left* to *right*, n = 30, 24, 12, 12, 18, 13, 18, 14, 18, and 12; n values correspond to separate experiments. Scatter plots are represented as mean ± SEM and were statistically analyzed using Kruskal–Wallis test with multiple comparisons (Dunn's test). ∗*p* < 0.05 and ∗∗*p* < 0.01, ∗∗∗*p* < 0.001, and ∗∗∗∗*p* < 0.0001 as compared with CCh-treated O1KO HEK-293 cells (*A*) or CCh-treated WT HEK-293 cells (*C*). ^$^*p* < 0.05, ^$$^*p* < 0.01, and ^$$$$^*p*< 0 .0001 as compared with their respective control (untreated) cells. HBS, Hepes-buffered saline; HEK-293, human embryonic kidney 293 cell line.

As reported previously, in O1KO cells, there is still a certain degree of NF-κB transcriptional activity upon stimulation with CCh, which was found to be entirely dependent on Ca^2+^ entry (Figs. 1*A* and 4A). Hence, we have performed further studies to identify the Ca^2+^-permeable channels involved in the remaining response to CCh in O1KO cells. We have tested the functional role of different channels involved in agonist-induced Ca^2+^ entry, such as TRPC1, TRPC6, Orai2, and Orai3, by inducing knockdown of these proteins using specific shRNA for TRPC1, TRPC6, and Orai3 or esiOrai2 (a heterogeneous mixture of siRNA that target Orai2 mRNA). As shown in Figure 5*A*, silencing of TRPC1 or Orai3 significantly attenuated CCh-evoked NF-κB transcriptional activity as compared with the response to CCh in O1KO cells transfected with scramble plasmid (*p* < 0.0001 and *p* < 0.001 for TRPC1 and Orai3, respectively). However, knockdown of TRPC6 (Fig. 5*A*) or Orai2 (Fig. S4) was without effect on CCh-evoked response. Attenuation of protein expression was confirmed by Western blotting (Fig. 5, *B* and *D*).

We further explored the role of Orai3 in CCh-evoked NF-κB activation using Orai1/2/3 triple-KO (TKO) cells. As shown in Figure 5*C*, and consistent with the results reported previously in Figure 1*A*, CCh was unable to fully activate NF-κB in O1KO cells. In these cells, knockdown of Orai3 by transfection with shOrai3 further attenuated CCh-evoked response, and similar results were observed in TKO cells, which further confirms that functional Orai3 plays a role in CCh-induced NF-κB transcriptional activity. It should be noted that knockdown of Orai2 was without effect on CCh-stimulated NF-κB activity (Fig. S4); therefore, the results observed in TKO cells should be attributed to Orai1 and Orai3. It is also important to mention that knockdown of Orai3 in WT HEK-293 cells was without effect on CCh-evoked NF-κB activation (Fig. 5*C*). Although speculative, the reason of the discrepant effects of Orai3 knockdown in WT and O1KO cells might reside in the predominant role of Orai1 in Ca^2+^ influx in WT cells, which might overcome the Orai3 deficiency. Protein expression was detected by Western blotting using the appropriate antibodies (Fig. 5, *C* and *D*). Altogether, these findings indicate that TRPC1 and Orai3 participate, together with Orai1α, which has a predominant role, in the activation of NF-κB by CCh.

### Orai1α modulates NF-κB transcriptional activity *via* PKCβ

**Figure 6:**
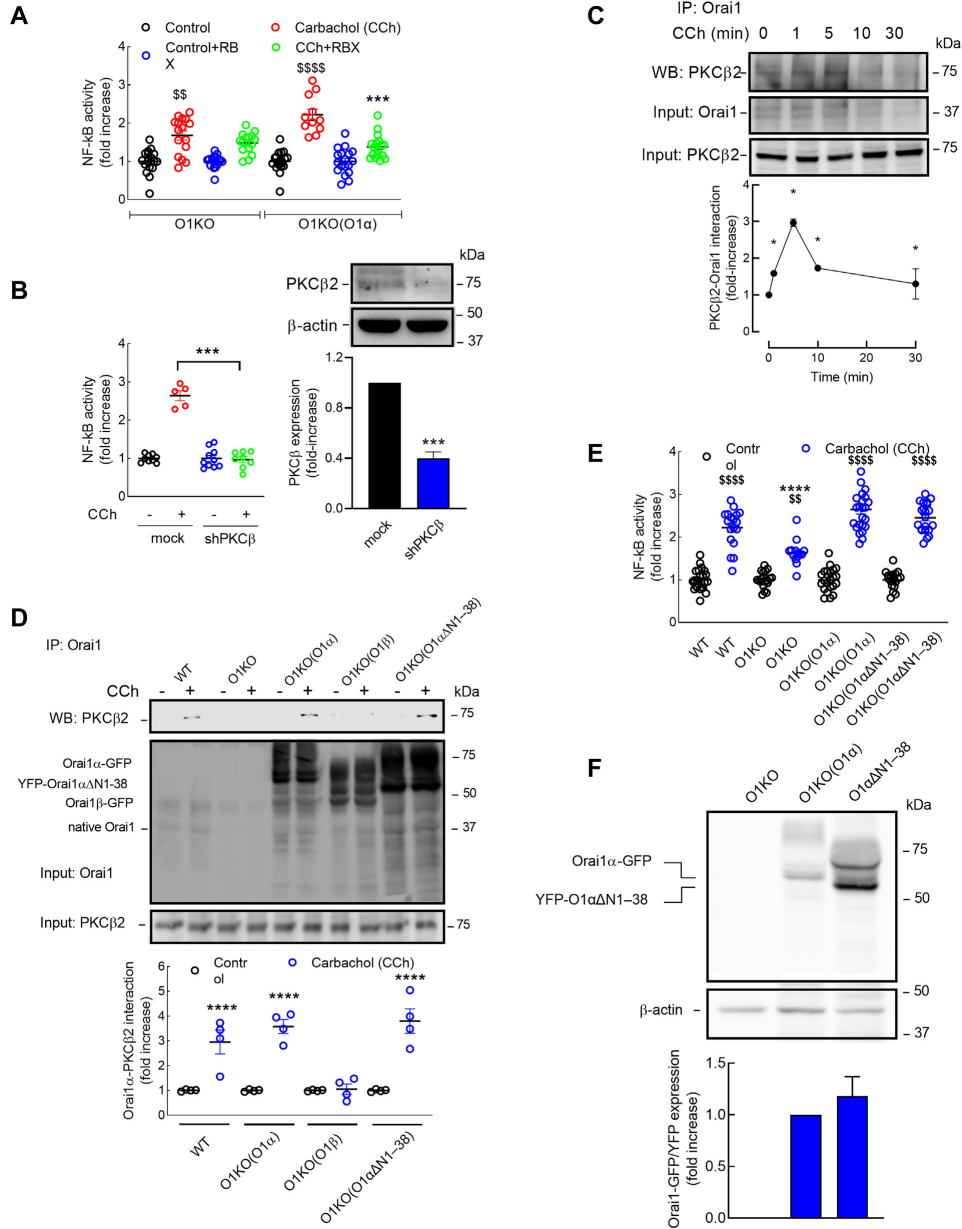
**PKCβ2 interacts with Orai1α and plays an essential role in agonist-stimulated NF-kB transcriptional activity.***A,* Orai1-KO HEK-293 cells (O1KO) transfected with either TK-promoter Orai1α (O1KO(O1α)) or scramble plasmid (O1KO) were transfected with pNL3.2.NFkB-RE[NlucP/NF-kB-RE/Hygro]. Forty-eight hours later, cells were suspended in HBS containing 1 mM Ca^2+^, pretreated with 20 nM ruboxistaurin (RBX) or the vehicle for 20 min, and then stimulated for 5 h with 100 μM CCh or the vehicle (control) and lysed. Luciferase activity was measured as described in the Experimental procedures section. Scatter plots are presented as mean ± SEM and were statistically analyzed using Kruskal–Wallis test with multiple comparisons (Dunn's test). ∗∗∗*p* < 0.001 as compared with CCh-treated O1KO(O1α) cells in the absence of RBX. ^$$^*p* < 0.001 and ^$$$$^*p* < 0.0001 as compared with their respective control (nonstimulated) cells. From *left* to *right*, n = 18, 16, 18, 15, 18, 12, 18, and 17; n values correspond to separate experiments. *B,* Orai1-KO HEK-293 cells expressing Orai1α were transfected with shPKCβ or empty vector (mock) as well as with pNL3.2.NFkB-RE[NlucP/NF-kB-RE/Hygro]. Forty-eight hours later, cells were suspended in HBS containing 1 mM Ca^2+^ and stimulated for 5 h with 100 μM CCh or the vehicle (control) and lysed. Luciferase activity was measured as described in the Experimental procedures section. Scatter plots are presented as mean ± SEM and were statistically analyzed using Kruskal–Wallis test with multiple comparisons (Dunn's test). ∗∗∗*p* < 0.001 as compared with CCh-treated mock cells. From *left* to *right*, n = 9, 5, 10, and 8; n values correspond to separate experiments. Cell lysates were also subjected to 10% SDS-PAGE and Western blotting with the anti-PKCβ2 antibody. Membranes were reprobed with anti-β-actin antibody for protein loading control. Blots are representative of three separate experiments, and intensity of PKCβ2 bands was normalized to β-actin and quantified (*bar graph*). *C,* WT HEK-293 cells were stimulated with 100 μM CCh for various periods (1–30 min) or left untreated and lysed. Cell lysates were immunoprecipitated with anti-Orai1 antibody. Immunoprecipitates were subjected to 10% SDS-PAGE and Western blotting with the anti-PKCβ2 antibody. Membranes were reprobed with the antibody used for immunoprecipitation for protein loading control. Alternatively, the cell lysates were subjected to 10% SDS-PAGE and subsequent Western blotting with anti-PKCβ2 antibody. Molecular masses indicated on the *right* were determined using molecular-mass markers run in the same gel. Data shown are presented as mean ± SEM of four independent experiments and are expressed as fold increase over the pretreatment level (experimental/control). *D,* WT HEK-293 cells (WT), Orai1-KO HEK-293 cells (O1KO), and Orai1-KO HEK-293 cells expressing either CMV-promoter Orai1α (O1KO(O1α)), Orai1β (O1KO(O1β)), or the N-terminal deletion mutant Orai1αΔN1–38 (O1αΔN1–38) together with STIM1 were stimulated for 5 min with 100 μM CCh in the presence of 1.8 mM extracellular Ca^2+^ and lysed. Cell lysates were immunoprecipitated with anti-Orai1 antibody. Immunoprecipitates were subjected to 10% SDS-PAGE and Western blotting with the anti-PKCβ2 antibody. Membranes were reprobed with the antibody used for immunoprecipitation for protein loading control. Alternatively, the cell lysates were subjected to 10% SDS-PAGE and subsequent Western blotting with anti-PKCβ2 antibody. Scatter plots are presented as mean ± SEM of four independent experiments and are expressed as fold increase over the pretreatment level (experimental/control). *E,* WT HEK-293 cells, Orai1-KO HEK-293 cells (O1KO), and Orai1-KO HEK-293 cells expressing either CMV-promoter Orai1α (O1KO(O1α)) or the N-terminal deletion mutant Orai1αΔN1–38 (O1αΔN1–38) together with STIM1 were transfected with pNL3.2.NFkB-RE[NlucP/NF-kB-RE/Hygro]. Forty-eight hours later, cells were suspended in HBS containing 1 mM Ca^2+^ and stimulated for 5 h with 100 μM CCh or the vehicle (control) and lysed. Luciferase activity was determined as described in the Experimental procedures section. Scatter plots are presented as mean ± SEM and were statistically analyzed using Kruskal–Wallis test with multiple comparisons (Dunn's test). ∗∗∗∗*p* < 0.0001 as compared with CCh-treated WT HEK-293 cells. ^$$^*p* < 0.001 and ^$$$$^*p* < 0.0001 as compared with their respective control (nonstimulated) cells. From *left* to *right*, n = 23, 18, 20, 14, 22, 24, 18, and 21; n values correspond to separate experiments. *F,* Orai1-KO HEK-293 cells (O1KO) and Orai1-KO HEK-293 cells expressing either CMV-promoter Orai1α (O1KO(O1α)) or the N-terminal deletion mutant Orai1αΔN1–38 (O1αΔN1–38) together with STIM1 were lysed, and cell lysates were subjected to 10% SDS-PAGE and Western blotting with the anti-Orai1 antibody. Membranes were reprobed with anti-β-actin antibody for protein loading control. Blots are representative of three separate experiments, and intensity of Orai1 bands was normalized to β-actin and quantified (*bar graph*). CCh, carbachol; CMV, cytomegalovirus; HBS, Hepes-buffered saline; HEK-293, human embryonic kidney 293 cell line; STIM, stromal interaction molecule; TK, thymidine kinase.

PKCβ is one of the “conventional” Ca^2+^ and diacylglycerol-regulated PKC isoforms that has been reported to be required for the recruitment of IKK into lipid rafts as well as the activation of IKK leading to the degradation of IκB and subsequent NF-κB activation (21, 22). To investigate the possible role of PKCβ in the activation of NF-κB by CCh mediated by Orai1α, O1KO cells were transfected with Orai1α, using expression plasmids carrying a TK promoter, and PKCβ functional role was prevented using the specific inhibitor ruboxistaurin (RBX) (23). As shown in Figure 6*A*, in O1KO cells, treatment with 20 nM RBX for 20 min did not significantly attenuate CCh-induced NF-κB transcriptional activity. Interestingly, inhibition of PKCβ by RBX significantly inhibited CCh-stimulated response in O1KO cells expressing Orai1α (Fig. 6*A*; *p* < 0.001), so that in the presence of RBX, the expression of Orai1α in O1KO cells was without effect on CCh-evoked NF-κB activation, which indicates that the functional role of Orai1α on CCh-stimulated NF-κB transcriptional activity strongly depends on PKCβ activation. These findings were confirmed by silencing PKCβ expression. As shown in Figure 6*B*, PKCβ knockdown in O1KO cells expressing Orai1α abolished CCh-evoked NF-κB activation, which further supports a functional role for PKCβ in Orai1α-dependent agonist–induced NF-κB activation.

PKCβ1 has been associated to Ca^2+^-dependent inactivation of Orai1-forming channels (24); meanwhile, SOCE has been reported to activate PKCβ2, which, in turn, modulates gene transcription through the transcriptional coactivators YAP/TAZ (25). Hence, we have further investigated the mechanism underlying the role of PKCβ2 on Orai1α-dependent NF-κB activation. As PKCβ is a Ca^2+^-activated PKC isoform, we have explored whether PKCβ2 is in close proximity and interacts with Orai1 by looking for coimmunoprecipitation between both proteins from cell lysates. Immunoprecipitation and subsequent SDS-PAGE and Western blotting were conducted using WT HEK-293 cells both at resting conditions and after stimulation with 100 μM CCh in the presence of 1 mM extracellular Ca^2+^. CCh stimulated a rapid coimmunoprecipitation between Orai1 and PKCβ2, which reached a maximum at 5 min and then decreased (Fig. 6*C*). After immunoprecipitation with the anti-Orai1 antibody, Western blotting revealed the presence of PKCβ2 in resting cells, which was transiently increased in CCh-treated cells (Fig. 6*C*, *top panel*; n = 4). Western blotting with anti-Orai1 antibody confirmed a similar content of this protein in all lanes (Fig. 6*C*, *bottom panel*). Next, we explored the possible interaction of PKCβ2 with the Orai1 variants by looking for coimmunoprecipitation in lysates from WT HEK-293 cells, O1KO cells, and O1KO cells expressing either Orai1α or Orai1β. As depicted in Figure 6*D*, *top panel*, treatment for 5 min with CCh (100 μM) significantly enhanced the interaction between PKCβ2 and either native Orai1 or Orai1α. Interestingly, we did not find enhanced interaction between both proteins in O1KO cells or in O1KO cells expressing Orai1β upon stimulation with CCh (Fig. 6*D*, *top panel*). It should be noted that we have detected a faint band in O1KO cells coimmunoprecipitated with anti-Orai1 antibody (Fig. 6*D*, *top panel*), which we have attributed to a nonspecific band, and has been subtracted to the results during the analysis. Western blotting with anti-Orai1 antibody confirmed the expression of this protein in all lanes, except in O1KO cells (Fig. 6*D*, *bottom panel*). These findings indicate that PKCβ2 exclusively interacts with Orai1α, which is consistent with the role of this variant in NF-κB activation.

It is well known that PKC binds AKAP79 at a site different from those bound by PKA or calcineurin. This interaction allows AKAP79 to spatially confine PKC to specific subcellular compartments modulating its function. In has recently been reported that the N terminus of Orai1 contains a region that interacts with AKAP79. This region, named AKAR, contains amino acids 39 to 59 (1) located within the N terminus of Orai1α exclusively. In order to further explore the mechanism of interaction between Orai1α and PKCβ2, we have indirectly tested the role of the AKAR region in this interaction in O1KO cells expressing an N-terminal deletion mutant Orai1αΔN1–38. As depicted in Fig. S5, CCh-induced Ca^2+^ mobilization was not statistically different in O1KO HEK-293 cells expressing CMV-promoter Orai1α or the N-terminal deletion mutant Orai1αΔN1–38 together with STIM1. We noticed that overexpression of Orai1α or the truncated construct and STIM1 modified the pattern of Ca^2+^ mobilization to a more sustained elevation in [Ca^2+^]_i_, which was slightly greater in cells expressing the Orai1αΔN1–38 mutant (Fig. S5). This is consistent with Orai1α having more Ca^2+^-dependent inactivation and the fact that the truncation used is missing Ser24 as previously described (2). As shown in Figure 6*D*, immunoprecipitation with the anti-Orai1 antibody followed by Western blotting with anti-PKCβ2 antibody revealed that treatment with 100 μM CCh for 5 min enhanced the interaction between Orai1αΔN1–38 and PKCβ2, reaching a value that was comparable to that observed in O1KO cells expressing full-length Orai1α (Fig. 6*D*, n = 4). These findings, together with the fact that Orai1β (lacking the N-terminal 64 amino acids of Orai1α) is unable to bind PKCβ2, strongly suggest that the AKAR region plays an essential role in the Orai1α–PKCβ2 association through AKAP79 anchoring interaction. Furthermore, our results indicate that CCh-induced NF-κB transcriptional activity was comparable in O1KO cells expressing either full-length Orai1α or Orai1αΔN1–38 mutant, which further suggests that the Orai1α AKAR region plays an important role in the activation of NF-κB (Fig. 6*E*). Western blotting of cell lysates with anti-Orai1 antibody confirmed the expression of full-length Orai1α and the Orai1αΔN1–38 mutant (Fig. 6*F*). Different bands were observed after Western blotting with the anti-Orai1 antibody, which is consistent with the presence of post-translational modified forms of Orai1 (27).

## Discussion

Our results reveal that agonist-stimulated NF-κB transcriptional activity is a Ca^2+^ influx–dependent cell process where Orai1 plays a predominant role, but it is also partially supported by TRPC1- and Orai3-mediated Ca^2+^ entry. Concerning the functional role of Orai1, two variants have been identified, Orai1α and Orai1β, which arises from alternative translation initiation from the same transcript, but only Orai1α plays a relevant role in agonist-induced NF-κB activation. We base this statement in different findings: First of all, Orai1α was able to rescue agonist-induced NF-κB activation in O1KO cells. By contrast, expression of Orai1β in O1KO cells did not significant modify agonist-evoked response. This difference cannot be attributed to a different efficiency in the expression of both Orai1 variants or to ectopic cellular location of Orai1β, as we found similar expression level and cellular localization of Orai1α and Orai1β in O1KO HEK-293 cells, and CCh-induced Ca^2+^ mobilization was also similar in Orai1α-expressing as well as Orai1β-expressing O1KO cells. Second, our results indicate that PKCβ is required for Orai1-dependent agonist-stimulated NF-κB transcriptional activity, and consistent with this, we have found that CCh induces rapid and transient interaction of PKCβ2 with Orai1α but not with Orai1β. These functional and biochemical findings provide strong evidence for a relevant role of Orai1α but not its variant, Orai1β, in agonist-induced NF-κB activation. Previous studies have also reported functional differences between both Orai1 variants. For instance, Orai1β less sensitive to Ca^2+^-dependent inactivation and Orai1α, but not Orai1β, supports arachidonate-regulated Ca^2+^ influx (19). Furthermore, we have recently reported that Orai1α modulates plasma membrane location and activation of TRPC1 channels upon Ca^2+^ store depletion (20). These findings suggest that the Orai1 variants exhibit differential properties and are nonredundant tailoring Ca^2+^ signals in response to physiological stimuli.

The role of Ca^2+^ entry *via* Orai1 in the activation of transcription factors, such as nuclear factor of activated T-cells, is well known and has been studied extensively; however, its role in the modulation of NF-κB transcriptional activity has been scarcely investigated. Previous studies have reported a role of extracellular Ca^2+^ influx *via* STIM1–Orai in T cells, a mechanism involving Ca^2+^-dependent PKCα-mediated phosphorylation of p65 (17). In these cells, the different dynamics of STIM1–Orai1-dependent Ca^2+^ signals generated by high-affinity and low-affinity antigens, ranging from sustained rises in [Ca^2+^]_i_ to Ca^2+^ oscillations, have been reported to be decoded by nuclear factor of activated T-cells and NF-κB, so that sustained [Ca^2+^]_i_ elevations activate both transcription factors, whereas Ca^2+^ oscillations and transient rises in [Ca^2+^]_i_ specifically activate NF-κB (28). In agreement with this, here we show that low agonist concentration, inducing predominantly Ca^2+^ oscillations, induces NF-κB activation, although with less efficacy than high agonist concentration, resulting in more sustained Ca^2+^ signals. Furthermore, previous studies have reported that silencing of Orai1 and Orai2 expression attenuate the Akt/mammalian target of rapamycin/NF-κB pathway in oral cancer cells, further supporting a role for Orai channels in NF-κB activation (29).

**Figure 7:**
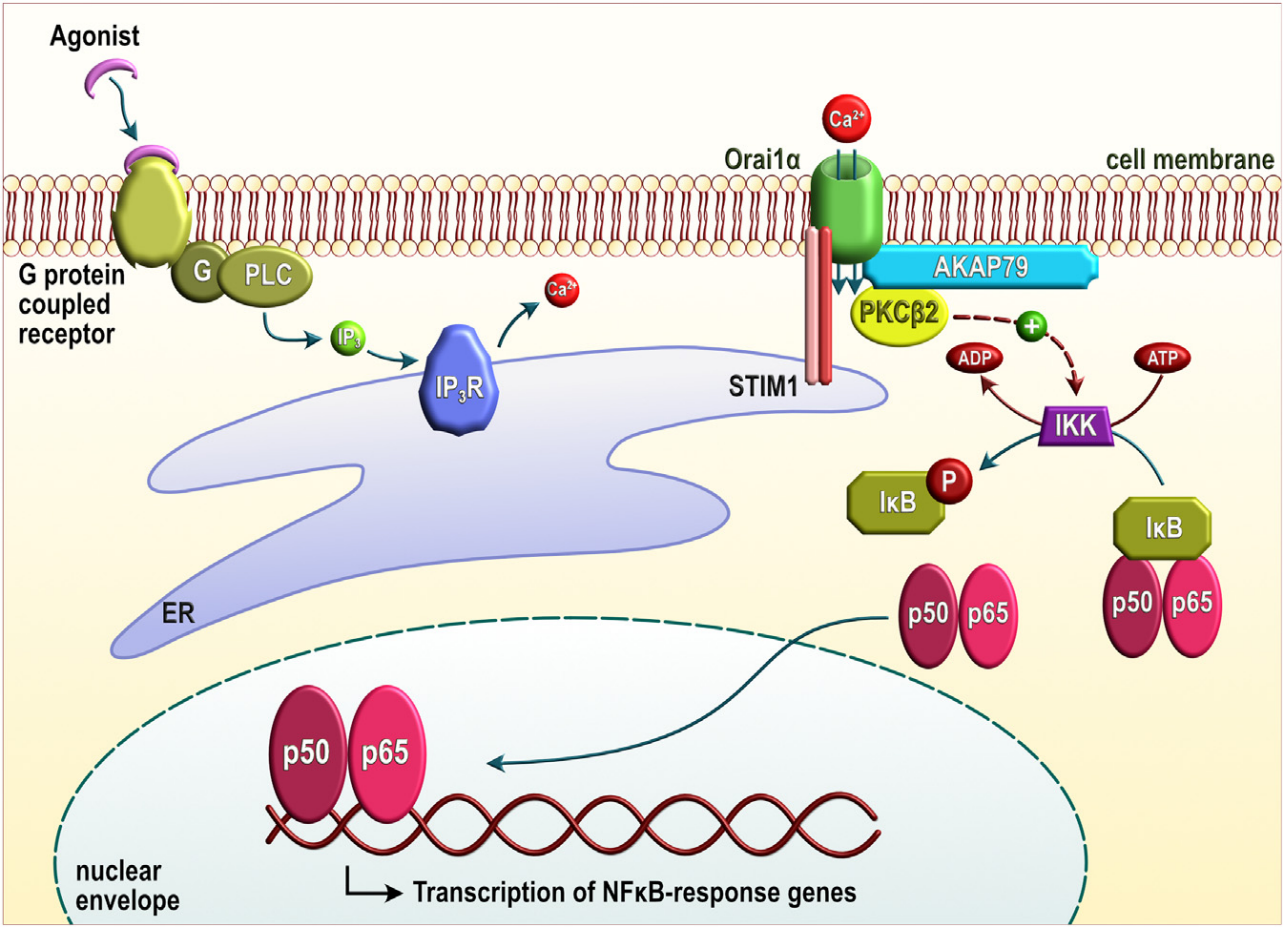
***Cartoon* summarizing Orai1α-dependent, agonist-stimulated, NF-κB transcriptional activity.** G protein–coupled receptor occupation leads to the activation of phospholipase C (PLC) that generates inositol 1,4,5-trisphosphate (IP_3_), which, in turn, induces Ca^2+^ release from the endoplasmic reticulum (ER). Ca^2+^ store depletion leads to Ca^2+^ influx *via* Orai1α, which enhances the Ca^2+^ concentration nearby. Orai1α and the Ca^2+^-regulated conventional PKC isoform, PKCβ2, are maintained in close apposition by the scaffold protein AKAP79. Therefore, the rise in cytosolic Ca^2+^ concentration nearby the channel results in activation of PKCβ2, which has been reported to lead to the activation of NF-κB *via* the classical IκB kinase (IKK)–IκB signaling pathway, thus, leading to IκB serine phosphorylation and proteasomal degradation, which releases active, dimeric, NF-κB that translocates to the nucleus and initiates transcription of NF-κB-responsive genes.

PKCα and PKCβ have been previously shown to be involved in Ca^2+^-dependent activation of NF-κB in lymphocytes (30, 31). They translocate to the plasma membrane upon receptor activation; however, the delay of the translocation varies from 10 min, for PKCα in T-cells (30), to 1 min for PKCβ in B-cells. Furthermore, Ca^2+^-binding affinities vary between the different conventional PKC isoforms, ranging from the submicromolar to the micromolar concentration (the Ca^2+^ concentration that yields half-maximal binding has been reported to be 0.7 ± 0.1 μM for PKCγ, 1.4 ± 0.1 μM for PKCα, and 5.0 ± 0.2 μM for PKCβ (32). Since PKCβ is the isoform with the lowest affinity for Ca^2+^, it is expected to be located in close proximity of the channel pore. Hence, altogether, we have focused on PKCβ as it rapidly translocates to the plasma membrane in response to Ca^2+^ signals (31) and because of its low Ca^2+^ affinity (32) might be located nearby the Orai1-forming channels. Our results indicate that PKCβ2 interacts with Orai1α upon agonist stimulation with a time course comparable to the translocation of PKCβ to the plasma membrane in B-cells upon BCR activation (31). Using an N-terminal deletion mutant of Orai1α lacking amino acids 1 to 38 (Orai1αΔN1–38), upstream of the AKAR (amino acids 39–59 (1)), our results suggest that the AKAR of Orai1α is essential for the interaction with PKCβ2. This is based on the observations that the interaction of full Orai1α and Orai1αΔN1–38 with PKCβ2 is similar, whereas Orai1β, lacking the N-terminal 64 amino acids, was unable to interact with PKCβ2. Consistent with this, agonist-stimulated NF-κB transcriptional activity was indistinguishable upon expression of Orai1αΔN1–38 or Orai1α in O1KO HEK-293 cells, whereas expression of Orai1β, lacking the AKAR, failed to rescue agonist-induced NF-κB activation in O1KO HEK-293 cells. These findings indicate that Orai1α interacts with PKCβ2 by a mechanism involving the N-terminal AKAR, which strongly suggests a role for the scaffolding protein AKAP79 in this process. AKAP79 has been reported to interact with PKC at a site distinct from those bound by PKA or calcineurin (26). Anchoring interaction of PKC with AKAP79 is relevant for a number of cellular processes, such as the modulation of adenylyl cyclase 2 activity, as well as the function of a variety of K^+^- and Ca^2+^-permeable channels (33, 34, 35). Altogether, our results suggest that the scaffolding protein AKAP79 might mediate the interaction between PKCβ2 and Orai1α and, upon agonist stimulation, Ca^2+^ influx through Orai1α leads to the rise of [Ca^2+^]_i_ nearby the channel pore, which, in turn, facilitates the activation of PKCβ2 and subsequent enhancement of the NF-κB transcriptional activity (Fig. 7). Our findings provide evidence that Orai1α and Orai1β might play differential functional roles in mammalian cells.

## Experimental procedures

### Reagents and antibodies

Fura-2 acetoxymethyl ester (AM) was from Molecular Probes. High-glucose Dulbecco’s modified Eagle’s medium, fetal bovine serum, trypsin, penicillin/streptomycin, TRIzol reagent, qRT–PCR primers, high-capacity complementary DNA reverse transcription kit, SYBR Green PowerUp, rabbit polyclonal anti-TRPC1 antibody (catalog number: PA577303, epitope: amino acids 557–571 of human TRPC1), Clean-Blot IP detection reagent, and SuperSignal West Dura extended duration substrate reagent, and Pierce BCA protein assay kit were purchased from Thermo Fisher Scientific. Complete EDTA-free protease inhibitor cocktail tablets were from Roche Diagnostics GmbH. DharmaFECT kb transfection reagent was obtained from Cultek. TG, histamine, protein A agarose beads, Hepes, EGTA, EDTA, bovine serum albumin (BSA), sodium azide, dimethyl-BAPTA, sodium ascorbate, RBX, esiRNA Orai2 (a heterogeneous mixture of siRNA that all target the same mRNA sequence), rabbit polyclonal anti-Orai1 antibody (catalog number: O8264, epitope: amino acids 288–301 of human Orai1), and rabbit polyclonal anti-β-actin antibody (catalog number: A2066, epitope: amino acids 365–375 of human β-actin) were obtained from Sigma. PKCβ shRNA (catalog number: sc-29450-SH; from Santa Cruz Biotechnology). Rabbit polyclonal anti-TRPC6 antibody (catalog number: TA328771, epitope: amino acid residues 573–586 of TRPC6) was from Origene Technologies, Inc. Rabbit monoclonal anti-PKCβ2 antibody (clone Y125, epitope located in the region near the C terminus of PKCβ2; catalog number: ab32026) and rabbit monoclonal anti-Orai3 antibody (clone EPR22575-17, catalog number: ab254260) were purchased from Abcam. Horseradish peroxidase–conjugated goat antimouse immunoglobulin G (IgG) antibody and goat anti-rabbit IgG antibody were from Jackson Laboratories. TK-promoter and CMV-promoter Orai1α-enhanced GFP (Egfp) and Orai1β-eGFP plasmids were kindly provided by Mohamed Trebak (Department of Pharmacology and Chemical Biology, University of Pittsburgh). pEYFP-Orai1α N-terminal deletion mutant (Orai1αΔN1–38) was kindly provided by Christoph Romanin (Institute of Biophysics, Johannes Kepler University Linz). shRNA-Orai3 was kindly provided by Rajender Motiani (Regional Centre for Biotechnology). Nano-Glo Luciferase Assay System and NanoLuc Reporter Vector with NF-κB Response Element were purchased from Promega. All other reagents were of an analytical grade.

### Cell culture and transfections

CRISPR-generated Orai1 single-KO HEK293 cells (O1KO), Orai1/2/3 TKO HEK-293 cells (TKO cells), and parental HEK-293 cells were kindly provided by Mohamed Trebak and cultured at 37 ºC with a 5% CO_2_ in high-glucose Dulbecco’s modified Eagle’s medium supplemented with 10% (v/v) fetal bovine serum and 100 U/ml penicillin and streptomycin, as described previously (36). For transient transfections, cells were grown to 60 to 80% confluency and transfected with TK-promoter Orai1α-eGFP or Orai1β-eGFP or CMV-promoter Orai1α-eGFP, Orai1β-eGFP, or pEYFP-Orai1αΔN1–38, depending on the experimental conditions, or with esiRNAOrai2 or nonspecific siRNA using DharmaFECT kb transfection reagent and were used 48 h after transfection. For NF-κB activity determination, cells (2 × 10^4^) were plated in 96-well microplates. For Western blotting and immunoprecipitation, cells (2 × 10^6^) were plated in 100 mm petri dish and cultured for 48 h, whereas, for Ca^2+^ imaging and confocal analysis, cells (4 × 10^5^) were seeded in a 35 mm 6-well multidish. In some experiments, cells were loaded with dimethyl BAPTA by incubation for 30 min with 10 μM dimethyl BAPTA/AM.

### NF-κB-luciferase reporter assay

WT HEK-293 cells, Orai1-KO HEK-293 cells, and Orai1-KO HEK-293 cells expressing either Orai1α or Orai1β were transfected with pNL3.2.NFkB-RE[NlucP/NF-kB-RE/Hygro] and 48 h later were stimulated in the absence or in the presence of CCh, as indicated. Luciferase activity of the lysates was measured using a Nano-Glo Luciferase Reporter Assay System, according to the manufacturer’s instructions, using a Varioskan Lux (Thermo Fisher Scientific).

### RNA extraction and real-time PCR

RNA extraction and qRT–PCR were performed by the Service of Techniques Applied to Bioscience of the University of Extremadura. Total RNA extraction was performed using TRIzol reagent according to the manufacturer’s specifications. The primers used are: hCOX-2 (forward primer: TCTGTACTGCGGGTGGAACA; reverse primer: CAATTTGCCTGGTGAATGATTC) and GAPDH (forward primer: CTAGGCGCTCACTGTTCTCTC; reverse primer: GTCCGAGCGCTGACCTT). The RNA was reverse transcribed using the high-capacity complementary DNA reverse transcription kit and SYBR Green qRT–PCR was performed using SYBR Green PowerUp in a QuantStudio 6 Flex Real-Time PCR System (Thermo Fisher Scientific). mRNA expression was calculated by the comparative CT (ΔΔCT) method using the formula RQ = 2^−ΔΔCT^. The amount of mRNA transcripts was normalized to GAPDH expression and represented as mean expression ± SEM.

### Immunoprecipitation and Western blotting

Immunoprecipitation and Western blotting were performed as described previously (37). Briefly, cells cultured on 100 mm petri dish (8 × 10^6^ cells) were stimulated with 100 μM CCh or with vehicle and subsequently lysed with ice-cold Nonidet P-40 buffer (pH 8) containing 137 mM of NaCl, 20 mM of Tris, 2 mM of EDTA, 10% glycerol, 1% Nonidet P-40, 1 mM of Na_3_VO_4_, and complete EDTA-free protease inhibitor tablets. Cell lysates (1 ml) were immunoprecipitated by incubation with 2 μg of anti-Orai1 antibody and 50 μl of protein A-agarose overnight at 4 °C on a rotary platform. Cell lysates and immunoprecipitates were resolved by 10% or 12% SDS-PAGE, and separated proteins were electrophoretically transferred onto nitrocellulose membranes for subsequent probing. Blots were incubated overnight with 10% (w/v) BSA in Tris-buffered saline with 0.1% Tween-20 (TBST) to block residual protein-binding sites. Immunodetection of Orai1 variants, β-actin, PKCβ2, TRPC1, TRPC6, and Orai3 was achieved by incubation for 1 h with anti-Orai1 antibody diluted 1:1000 in TBST, 1 h with anti-β-actin antibody diluted 1:2000 in TBST, 1 h with anti-PKCβ2 or anti-Orai3 antibody diluted 1:500 in TBST, or overnight with anti-TRPC1 and TRPC6 diluted 1:1000 in TSBT. The primary antibody was removed, and blots were washed six times for 5 min each with TBST. To detect the primary antibody, blots were incubated for 1 h with horseradish peroxidase–conjugated goat antimouse IgG antibody, horseradish peroxidase–conjugated goat anti-rabbit IgG antibody diluted 1:10,000 in TBST, or Clean-Blot IP Detection Reagent diluted 1:250 in TBST, and then exposed to enhanced chemiluminescence reagents for 5 min. The antibody binding was assessed with a ChemiDoc Imaging System (Bio-Rad), and the density of bands was measured using ImageJ software, v.1.8.0_172 (National Institutes of Health). Data were normalized to the amount of protein recovered by the antibody used for the immunoprecipitation or to β-actin from the same gel.

### Determination of cytosolic free-Ca^2+^ concentration ([Ca^2+^]_i_)

Cells were loaded with fura-2 by incubation with 5 μM fura-2/AM for 30 min at 37 °C. Coverslips with cultured cells were mounted on a perfusion chamber and placed on the stage of an epifluorescence inverted microscope (Nikon Eclipse Ti2) with an image acquisition and analysis system for videomicroscopy (NIS-Elements Imaging Software, version 5.02.00; Nikon). Cells were continuously superfused at room temperature with Hepes-buffered saline containing (in millimolar) 125 NaCl, 5 KCl, 1 MgCl_2_, 5 glucose, and 25 Hepes, pH 7.4, supplemented with 0.1% (w/v) BSA. Cells were examined at 40× magnification (Nikon CFI S FLUOR 40× Oil) and alternatively excited with light from a xenon lamp passed through a high-speed monochromator Optoscan ELE 450 (Cairn Research) at 340/380 nm. Fluorescence emission at 510 nm was detected using a cooled digital sCMOS camera PCO Panda 4.2 (Excelitas PCO GmbH) and recorded using NIS-Elements AR software (Nikon). Fluorescence ratio (F340/F380) was calculated pixel by pixel, and the data were presented as ΔF_340_/F_380_. CCh-evoked changes in [Ca^2+^]_i_ were estimated as the area under the curve measured as the integral of the rise in fura-2 fluorescence ratio 10 min after the addition of the agonist and taking a sample every second.

### Analysis of Ca^2+^ oscillations

The analysis of Ca^2+^ oscillations was performed as described previously (10). All Ca^2+^ traces obtained in the Ca^2+^-imaging experiments were plotted using GraphPad Prism, v.8.4.3 (GraphPad Software, Inc). The number of oscillations per 8 min were counted by the function signal.find_peaks from the SciPy 1.8.1 library for Python 3.9. Then, cells were classified in three groups, and a percentage of each group of cells was calculated for each individual coverslip. The first group, oscillating cells, includes cells showing regenerative oscillations after CCh stimulation, where each oscillation returns to baseline before the start of the next oscillation. A second group, nonoscillating cells, includes cells showing a sustained or transient cytosolic Ca^2+^ signal for at least 3 min after stimulation. Finally, the last group includes cells that show either no response to CCh stimulation or showed only one initial spike and remained at baseline for the duration of the recording.

### Confocal microscopy analysis

Cells were seeded in 35 mm six-well multidish and transfected with the indicated plasmids. Cells were imaged 48 h post-transfection using a confocal microscope (LSM900; Zeiss) with 63× oil immersion objective, using an image acquisition and analysis system for video microscopy (ZEN Software; Zeiss).

### Statistical analysis

All data are presented as the mean ± SEM. Analysis of statistical significance was performed using GraphPad Prism, version 8.4.3. Kruskal–Wallis test combined with Dunn's post hoc test were used to compare the different experimental groups. For comparison between two groups, the Mann–Whitney *U* test was used. All data with *p* < 0.05 were deemed significant; “ns” = nonsignificant.

## Data availability

All the data used are provided in this article or the supporting information.

## Ethics approval and consent to participate

Experimental procedures were approved by the local ethical committee (University of Extremadura and Extremadura Health Service).

## Supporting information

This article contains supporting information.

## Conflict of interest

The authors declare that they have no conflicts of interest with the contents of this article.
