# Supplementary material for: The store-operated Ca2+ channel Orai1α is required for agonist-evoked NF-κB activation by a mechanism dependent on PKCβ2
Source: J Biol Chem. 2023 Jan 7;299(2):102882. doi: 10.1016/j.jbc.2023.102882 (PMC9922819; doi:10.1016/j.jbc.2023.102882)
Supplement: Supplemental data [file mmc1.docx]

Supplementary material

The store-operated Ca^2+^ channel Orai1α is required for agonist-evoked NF-κB activation by a mechanism dependent on PKCβ2

## Joel Nieto-Felipe^†^, Jose Sanchez-Collado^†^, Isaac Jardin, Gines M. Salido, Jose J. Lopez*, Juan A. Rosado*

Department of Physiology (Cellular Physiology Research Group), Institute of Molecular Pathology Biomarkers (IMPB), University of Extremadura, 10003-Caceres, Spain.

*Corresponding author. Email: [jarosado@unex.es](mailto:jarosado@unex.es) and jjlopez@unex.es

**This PDF file includes:**

Fig. S1-S5

**Figure S1. TG and TNFα promote an increase in COX2 mRNA expression in HEK-293.** RT-qPCR expression analysis of COX-2 mRNA transcripts in control and stimulated cells for 5h with 1µM TG or 20ng/µL TNFα. Values were normalized to GADPH mRNA expression and presented as fold-change (experimental/control) and expressed as mean ± S.E.M. of 6 separate experiments. Data were statistically analyzed using Kruskal–Wallis test with multiple comparisons (Dunn’s test) (*** *p* < 0.001 as compared to control cells).


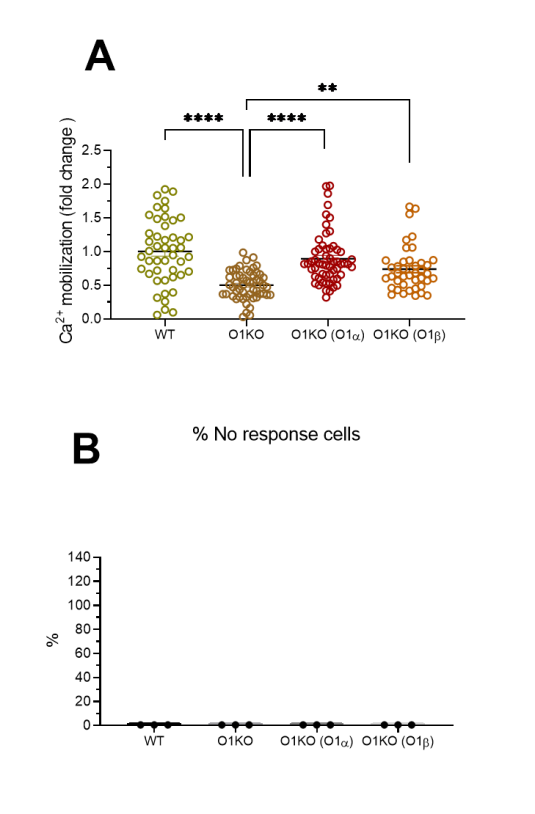


**Figure S2. Role of Orai1α and Orai1β in carbachol-induced Ca^2+^ mobilization.** Wild type HEK-293 cells (WT), Orai1-KO HEK-293 cells (O1KO) and Orai1-KO HEK-293 cells expressing either Orai1α (O1KO(O1α)) or Orai1β (O1KO(O1β)) were superfused with HBS containing 1 mM Ca^2+^ and stimulated with 100 µM CCh (as described in legend to Figure 2). *A***,** Quantification of Ca^2+^ mobilization for all the conditions estimated in all the cells. *B***,** Quantification of the percentage of non-responding cells (n = 3; n-values correspond to independent experiments). Scatter plots are represented as mean ± SEM and were statistically analyzed using Kruskal–Wallis test with multiple comparisons (Dunn´s test). ***p* < 0.01 and *****p*<0.0001).


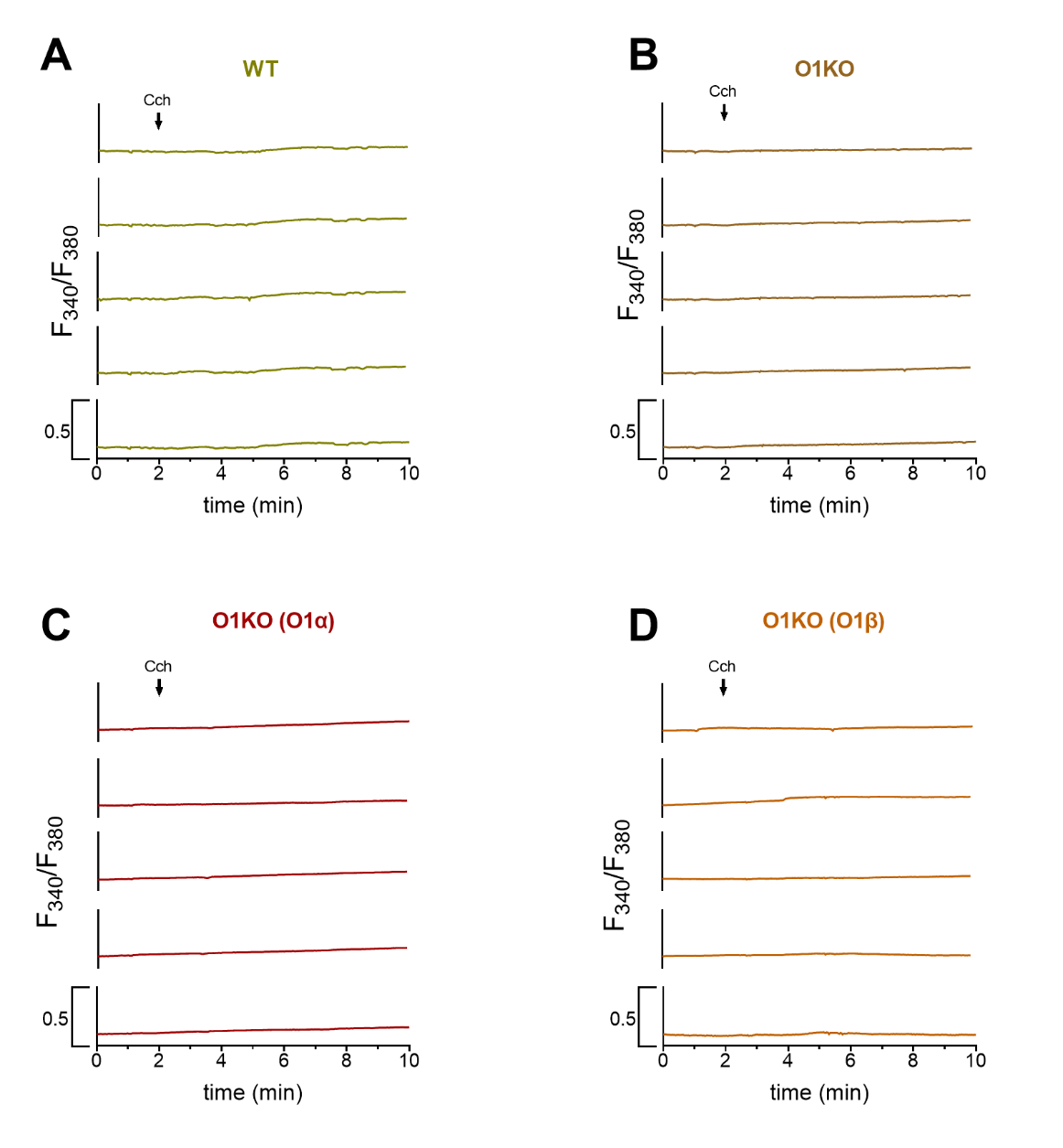


**Figure S3. Ca^2+^ mobilization in BAPTA-loaded cells.** Representative Ca^2+^ mobilization in response to 100 µM carbachol (CCh) measured using fura-2 in wild type HEK-293 cells (WT, *A*), Orai1-KO HEK-293 cells (O1KO; *B*) and Orai1-KO HEK-293 cells expressing either Orai1α (O1KO(O1α); *C*) or Orai1β (O1KO(O1β); *D*), as described. Cells were superfused with Ca^2+^-free HBSS (100µM EGTA added) and stimulated with 100 µM CCh at 2 min (indicated by arrow). Representative traces from five cells/condition were chosen to represent the datasets.


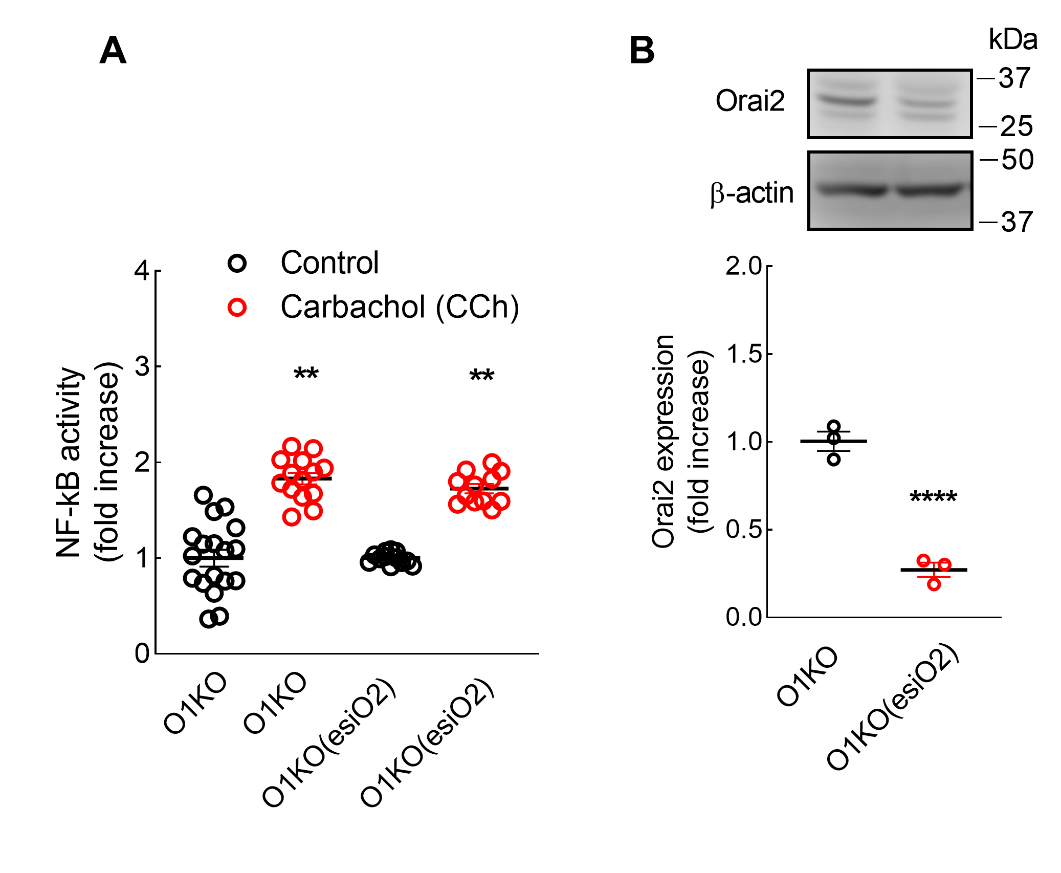


**Figure S4. Orai2 is not involved in carbachol-evoked NF-κB activation in Orai1-KO cells.** *A*, Orai1-KO HEK-293 cells (O1KO) transfected with either esiRNA Orai2 (O1KO(esiO2)) or scramble plasmid (O1KO) were transfected with pNL3.2.NFkB-RE[NlucP/NF-kB-RE/Hygro]. Forty-eight hours later cells were suspended in HBS containing 1 mM Ca^2+^ and then stimulated for 5h with 100 µM CCh or the vehicle (Control) and lysed. Luciferase activity of the lysates was measured using a Nano-Glo Luciferase Reporter Assay System, according to the manufacturer’s instructions. From left to right, n= 18, 14, 12 and 12. *B*, Orai1-KO HEK-293 cells (O1KO) transfected with either esiRNA Orai2 (O1KO(esiO2)) or scramble plasmid (O1KO). Forty-eight hours later cells were lysed and whole cell lysates were subjected to 10% SDS-PAGE and Western blotting with the anti-Orai2 antibody, as described in Experimental procedures. Membranes were reprobed with anti-β-actin antibody for protein loading control. Molecular masses indicated on the right were determined using molecular-mass markers run in the same gel. Blots are representative of three separate experiments. Scatter plots are represented as mean ± SEM and were statistically analyzed using Kruskal–Wallis test with multiple comparisons (Dunn´s test; *A*) or with the Mann–Whitney U test (*B*). ***p* < 0.01 and **** *p* < 0.001 as compared to their respective control.


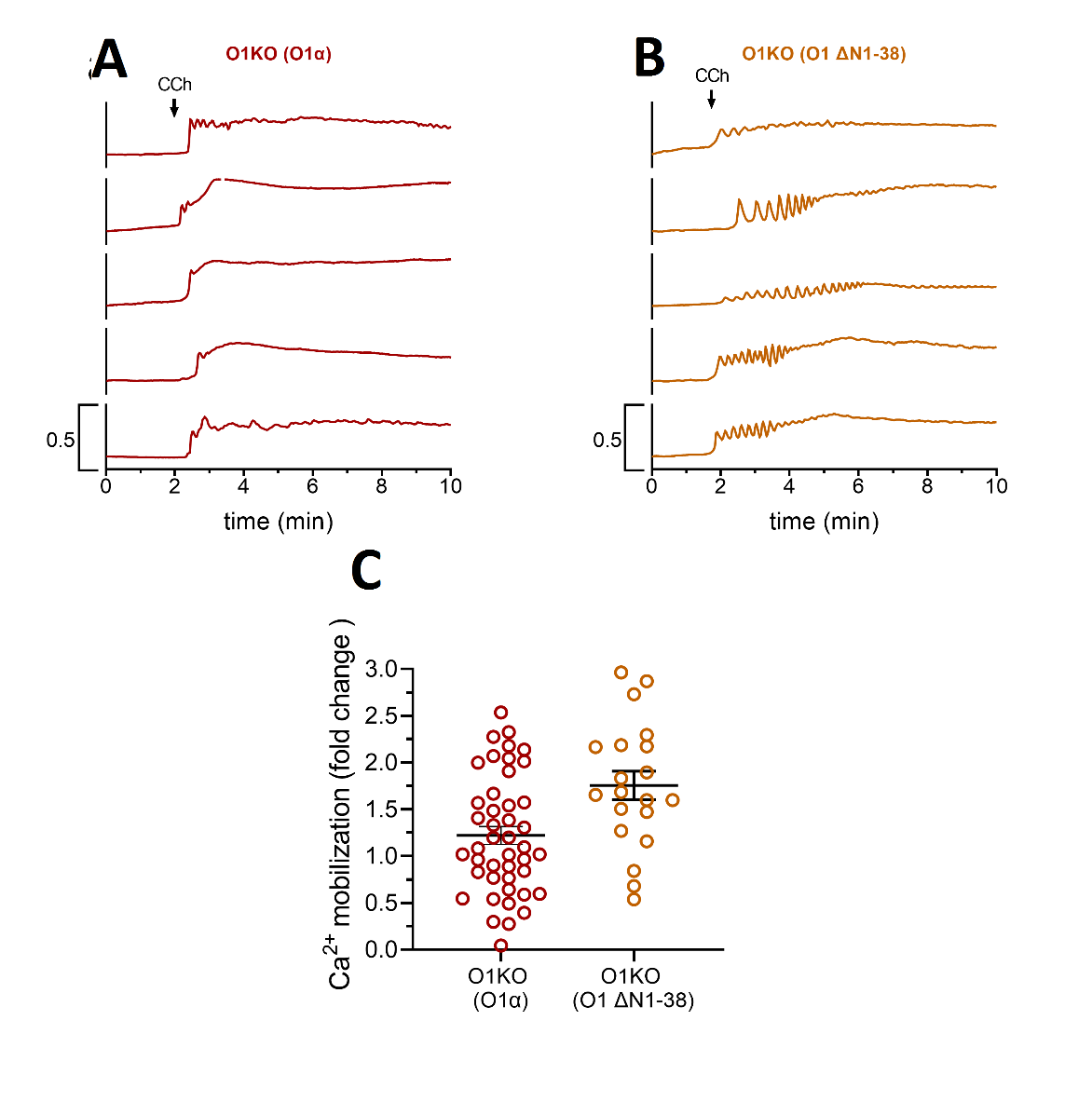


**Figure S5. Carbachol-induced Ca^2+^ mobilization in cells expressing Orai1α or the N-terminal deletion mutant Orai1αΔN1–38.** *A-B*, Representative Ca^2+^ mobilization in response to 100 µM CCh measured using fura-2 in Orai1-KO HEK-293 cells expressing either Orai1α (O1KO(O1α)) or the N-terminal deletion mutant Orai1αΔN1–38 (O1KO (O1ΔN1–38)), as described. Cells were superfused with HBS containing 1 mM Ca^2+^ and stimulated with 100 µM CCh at 2 min (indicated by arrow). Representative traces from five cells/condition were chosen to represent the datasets. *C*, Quantification of Ca^2+^ mobilization for both conditions estimated in all the cells. Scatter plots are represented as mean ± SEM and were statistically analyzed using Mann–Whitney U test. From left to right, n=44 and 20; n-values correspond to individual cells).
